# Cytosolic Phospholipase A2 Alpha Regulates TLR Signaling and Migration in Metastatic 4T1 Cells

**DOI:** 10.3390/ijms20194800

**Published:** 2019-09-27

**Authors:** Hanna Maja Tunset, Astrid Jullumstrø Feuerherm, Linn-Karina Myrland Selvik, Berit Johansen, Siver Andreas Moestue

**Affiliations:** 1 Department of Circulation and Medical Imaging, Faculty of Medicine, Norwegian University of Science and Technology, P.O. Box 8905, 7491 Trondheim, Norway; siver.a.moestue@ntnu.no; 2 Center for Oral Health Services and Research (TkMidt), 7030 Trondheim, Norway; astrid.j.feuerherm@ntnu.no; 3 Department of Biology, Norwegian University of Science and Technology, Realfagbygget, 7491 Trondheim, Norway; linn-karina.m.selvik@ntnu.no; 4 Department of Health Sciences, Nord University, P.O. Box 1490, 8049 Bodø, Norway

**Keywords:** eicosanoids, cPLA2 inhibitor, migration, metastasis, 4T1, 67NR, triple-negative breast cancer, Toll-like receptor

## Abstract

Metastatic disease is the leading cause of death in breast cancer patients. Disrupting the cancer cell’s ability to migrate may be a strategy for hindering metastasis. Cytosolic phospholipase A2 α (cPLA2α), along with downstream proinflammatory and promigratory metabolites, has been implicated in several aspects of tumorigenesis, as well as metastasis, in various types of cancer. In this study, we aim to characterize the response to reduced cPLA2α activity in metastatic versus non-metastatic cells. We employ an isogenic murine cell line pair displaying metastatic (4T1) and non-metastatic (67NR) phenotype to investigate the role of cPLA2α on migration. Furthermore, we elucidate the effect of reduced cPLA2α activity on global gene expression in the metastatic cell line. Enzyme inhibition is achieved by using a competitive pharmacological inhibitor, cPLA2α inhibitor X (CIX). Our data show that 4T1 expresses significantly higher cPLA2α levels as compared to 67NR, and the two cell lines show different sensitivity to the CIX treatment with regards to metabolism and proliferation. Inhibition of cPLA2α at nontoxic concentrations attenuates migration of highly metastatic 4T1 cells, but not non-metastatic 67NR cells. Gene expression analysis indicates that processes such as interferon type I (IFN-I) signaling and cell cycle regulation are key processes regulated by cPLA2a in metastatic 4T1 cells, supporting the findings from the biological assays. This study demonstrates that two isogenic cancer cell lines with different metastatic potential respond differently to reduced cPLA2α activity. In conclusion, we argue that cPLA2α is a potential therapeutic target in cancer and that enzyme inhibition may inhibit metastasis through an anti-migratory mechanism, possibly involving Toll-like receptor signaling and type I interferons.

## 1. Introduction

Breast cancer is the most common cancer in women, causing over half a million deaths per year worldwide [1]. Most of these patients die of metastasis, and not of the primary tumor [2]. Thus, preventing cancers from developing metastatic lesions can save the lives of numerous patients. Metastasis consists of multiple steps that the cells from the primary tumor need to execute in order to complete the formation of a distant lesion. Stopping cells from undergoing one or more of these steps may represent a therapeutic opportunity to stop metastasis.

Inflammation and cancer are tightly connected. Immunocompetent cells and tumorigenic cells can operate through the same signaling pathways and the inflammasome has emerged as a potential target to combat cancer [3]. A fundamental attribute of the metastatic cancer cell is the ability to migrate through tissue. Inflammatory signaling is a central component of induction of the migratory phenotype in nonmalignant as well as malignant conditions [4]. A key regulatory step of inflammation is the formation of small auto- and paracrine bioactive lipids. The phospholipase A2 enzymes constitute a superfamily of lipolytic enzymes that catalyze the hydrolysis of membrane phospholipids yielding lysophospholipids and free fatty acids [5]. In contrast to other phospholipase A2 enzymes (i.e., group II sPLA2, group V sPLA2, group VI iPLA2, and more), the group IVA cytosolic PLA2 (cPLA2α or PLA2 GIVA, gene PLA2G4A) is highly selective for arachidonic acid (AA) in the phospholipid *sn*-2 position. Therefore, it is considered to be the most important rate-limiting factor in the formation of AA-derived bioactive lipids, which play important roles in the microenvironment both in cancer and inflammatory diseases [6]. When cPLA2α hydrolyzes an intracellular membrane phospholipid that contains esterified arachidonic acid (AA), a free AA molecule and a lysophospholipid are generated [5]. Further metabolization by downstream enzymes, such as cyclooxygenase 2 (COX-2) or lipoxygenases, results in the generation of a spectrum of lipid-derived signaling mediators, such as prostaglandins and leukotrienes [7]. The activity of pathways, such as Raf/Ras/MEK/Erk, may lead to the activation of cPLA2α by phosphorylation, and translocation to membranes is stimulated by increased intracellular Ca^2+^ [7]. The cPLA2α signaling can interact with oncogenic pathways, such as the (PI3K)/Akt pathway and nuclear factor kappa B (NF-κB) pathway [8]. Accordingly, cPLA2α has been implicated in tumorigenesis, cancer progression, and metastasis in several cancer forms [9,10,11,12,13,14,15], including breast cancer [16,17,18,19,20,21,22]. In breast cancer, high cPLA2α expression levels are associated with the more aggressive, triple-negative phenotypes [18] and lower survival rates [17], suggesting a role in breast cancer metastasis. This effect may be mediated by prostaglandin E2 (PGE2), which is a known tumorigenic and promigratory eicosanoid produced from AA [13,23,24,25].

For a long time, inhibition of cPLA2α has been proposed as a promising anti-inflammatory target [26]. Furthermore, cPLA2α inhibition also has shown anti-tumorigenic and anti-angiogenic effects in cancer [13,20]. We have previously shown that various cPLA2α inhibitors efficiently target inflammation, tumor progression, and angiogenesis, in vitro and in vivo [20,27,28,29,30]. In this work, the low-molecular cPLA2α inhibitor X (CIX) (Coegin Pharma AS, Trondheim, Norway) was applied to investigate the significance of the enzyme in metastatic and non-metastatic murine breast cancer cell lines. CIX is a synthetic ω3-polyunsaturated fatty acid derivative which potently and selectively inhibits cPLA2α in vitro and in vivo [31]. In an isogenic cell line pair, the impact of cPLA2α inhibition on both viability, proliferation, and migration was investigated. A global gene expression analysis was performed to characterize the effects of cPLA2α on a signaling network level. Thus, this study contributes novel information on how cPLA2α inhibition differentially affects phenotypic changes at the transcriptome level in a metastatic cancer cell line, implying that cPLA2α may be a therapeutic anti-metastatic target.

## 2. Results and Discussion

### 2.1. Metastatic 4T1 Cells Express Higher Levels of cPLA2α than Non-Metastatic 67NR Cells

The 4T1 model consists of several isogenic cell lines that arise from the same murine breast tumor, but have widely different metastatic abilities [32]. We used two cell lines from the 4T1 model, 67NR, and 4T1, that are non-metastatic and highly metastatic, respectively [32], to investigate whether cPLA2α expression and activity could be involved in the metastatic phenotype.

**Figure 1:**
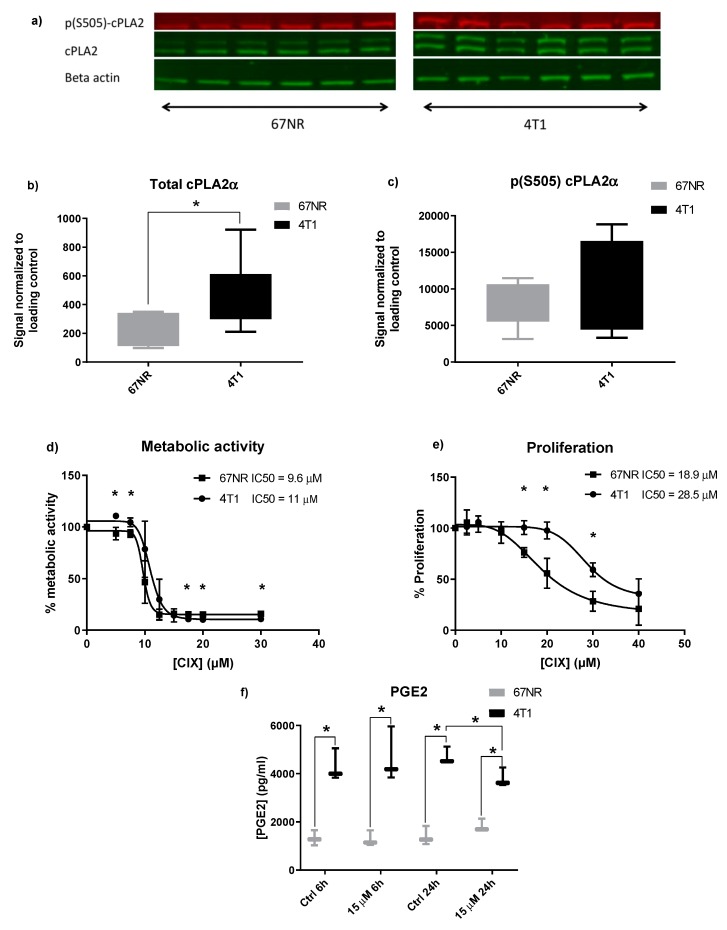
Response to cytosolic phospholipase A2 α (cPLA2α) inhibitor X (CIX) and expression of cPLA2α in 67NR and 4T1. (**a**) Image showing bands for basal p-cPLA2α, cPLA2α, and beta-actin expression in 67NR and 4T1 cells on the same membrane. (**b**) Box and whisker plots (min-max) of total cPLAα protein normalized to beta-actin. (**c**) Box and whisker plots (min-max) of phosphorylated cPLAα (p-S505) protein normalized to beta-actin. (**d**) Viability curve based on XTT (2,3-bis-(2-methoxy-4-nitro-5-sulfophenyl)-2H-tetrazolium-5-carboxanilide) metabolization. Points represent grand mean of *n* = 3 experiments with *n* = 6 technical replicates per condition. IC50 values (48 h) are given in μM. * *p* ˂ 0.05 4T1 vs. 67NR at the same concentration. (**e**) Proliferation curve based on EdU incorporation. Points represent grand mean of *n* = 3 experiments with *n* = 6 technical replicates per condition. IC50 values (24 h) are given in μM. * *p* ˂ 0.05 4T1 vs. 67NR at same concentration. (**f**) PGE2 levels in 67NR and 4T1 at 6 h and 24 h of treatment. The box and whisker plot (min-max) is based on *n* = 3 experiments with *n* = 3 technical replicates per condition. * *p* ˂ 0.05 4T1 vs. 67NR.

Initially, the baseline expression of cPLA2α protein in both cell lines was determined by Western blotting (Figure 1a–c). Two bands were observed for total protein detection, as is often observed for cPLA2α blots [33] with the slightly larger band being more intense in 4T1 (Figure 1a). The 4T1 cells expressed 1.95-fold more cPLA2α protein than 67NR cells (Figure 1b). Phosphorylation of cPLA2α on S505 typically corresponds to activation of cPLA2α [7], and a nonsignificant tendency of higher phosphorylation status was seen in 4T1 (Figure 1c). In addition to more total protein, this may imply that 4T1 has higher basal activity in pathways involving cPLA2α. This is in line with previous findings on cPLA2α in patient samples, where higher expression of cPLA2α has been measured in metastases as compared with primary tumor within the same patient [22].

### 2.2. Non-Metastatic 67NR and Metastatic 4T1 Cells Show Different Sensitivity to CIX Treatment

Next, we employed the cPLA2α inhibitor CIX to assess the effect of cPLA2α inhibition in our model. In order to establish a dose-response relationship of CIX and viability, we tested a range of doses on both cell lines in metabolic activity (XTT) and proliferation (EdU incorporation) assays. Both cell lines displayed a dose-dependent relationship between CIX concentration and assay output (Figure 1d–e). In the XTT assay, 67NR and 4T1 displayed IC50 values of 9.6 µM and 11 µM, respectively, after 48 h. The difference was not statistically significant (*p* < 0.05) at 10 µM, likely due to higher inter-assay variation, there was, however, a significant difference at lower and higher doses. For proliferation, the IC50 values were 18.9 µM and 28.5 µM (67NR and 4T1, respectively) after 24 h.

The ability of the cPLA2α inhibitor CIX to impede proliferation differed greatly in the two cell lines. Hence, lower mitochondrial metabolism did not directly correspond to lowered proliferation. As 4T1 has a more metabolically adaptive nature than 67NR [34], it may be that 4T1 is more robust to these effects and will continue proliferating even under poor conditions such as compromised mitochondrial metabolism. This is also in line with the observation that 4T1 has a shorter doubling time than 67NR and is generally less sensitive to stressors (our observations). Interestingly, an upregulation of cPLA2α protein in response to doxorubicin in breast cancer cell lines and patients has been reported, and combination treatment of cPLA2α inhibitors or siRNA augmented the antiproliferative effect of doxorubicin [35]. This indicates that cPLA2α plays an important role in regulating the viability and proliferation of breast cancer cells and may suggest that cells with higher cPLA2α expression have a survival advantage over lower cPLA2α expressing cells.

### 2.3. Inhibition of cPLA2α Reduces PGE2 Production in Metastatic 4T1 Cells

PGE2, a tumorigenic and promigratory metabolite downstream of cPLA2α and COX-2, was measured in both cell lines after treatment with 7.5 and 15 µM CIX for 6 and 24 h (Figure 1f). PGE2 levels were significantly higher in 4T1 as compared to 67NR at both time points (3.25-fold and 3.38-fold difference at 6 h and 24 h, respectively, Figure 1f), reflecting higher expression and activity of cPLA2α, which may also be related to increased migratory potential of metastatic cells [25]. Treatment with 15 µM CIX significantly lowered PGE2 in 4T1 after 24 h (0.81-fold), whereas no reduction was observed in 67NR. This may imply that CIX normalizes PGE2 levels by interrupting cPLA2α dependent AA-release, but PGE2 is not completely depleted. Other PLA2 isoenzymes contribute to AA accumulation as part of their unsaturated fatty acid release, thereby maintaining basal PGE2 levels [36].

### 2.4. cPLA2α Inhibition Impedes Migration in Metastatic 4T1 Cells

**Figure 2:**
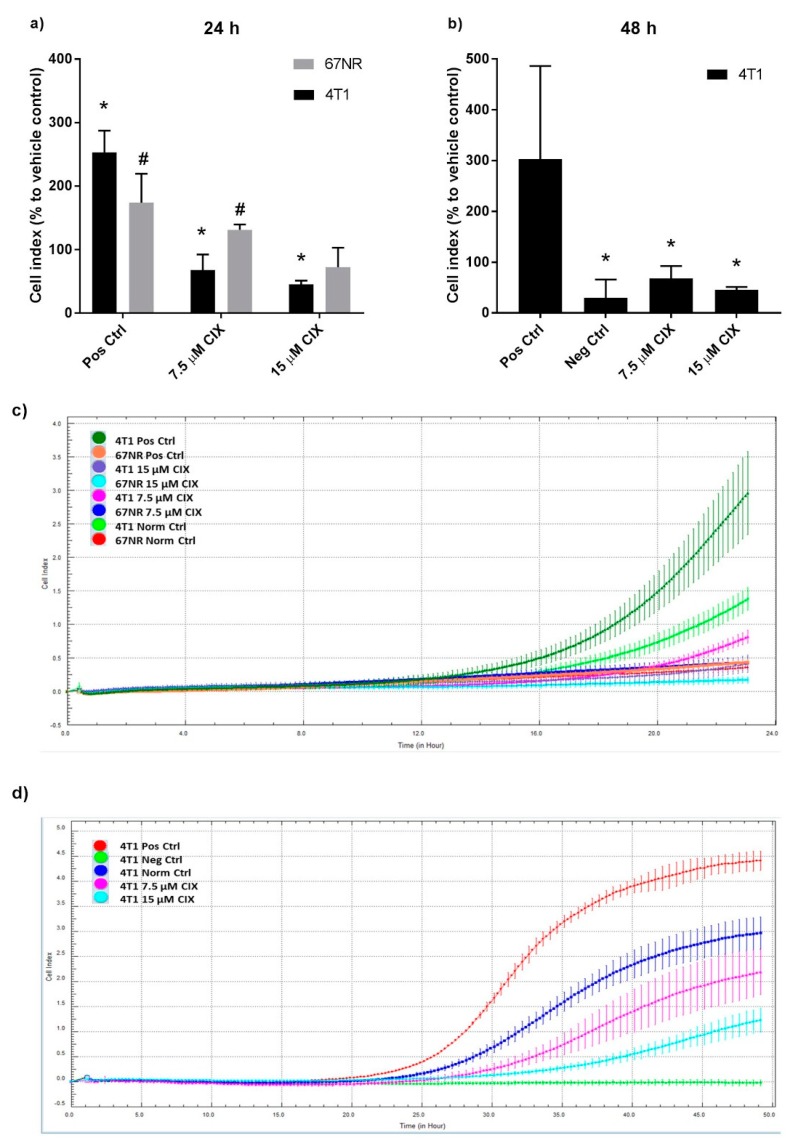
CIX inhibits migration in 4T1 cells, but not in 67NR cells. (**a**) Cell index (CI) related to vehicle control at the end of the 24 h treatment. Bars show the percentage mean ± SD of 3 experiments, normalized to vehicle control within experiment, *n* = 4 technical replicates per experiment (**b**) CI related to vehicle control at the end of 48 h treatment. Bars show the percentage mean ± SD of 3 experiments, normalized to vehicle control within experiment, *n* = 2 to 4 technical replicates per experiment. (**c**) CI curves for 67NR and 4T1 over 24 h. Each curve shows mean CI ± SD of *n* = 4 technical replicates. Representative experiment of *n* = 3 distinct experiments. (**d**) CI curves for 4T1 over 48 h. Each curve shows mean CI ± SD of *n* = 4 technical replicates for CIX treated groups and Norm Ctrl, *n* = 2 technical replicates for Pos Ctrl and Neg Ctrl. Representative experiment of *n* = 3 distinct experiments. (**a–d**) Norm Ctrl: Normal (vehicle) control. Norm Ctrl and all treated samples were subjected to 5% FBS in lower wells. Pos Ctrl: Positive control (high chemoattractant, 10% FBS in lower wells). Neg Ctrl: Negative control (0.5% FBS in lower wells). * *p* ˂ 0.05 vs. 4T1 Norm Ctrl, # *p* ˂ 0.05 vs. 67NR Norm Ctrl.

As cPLA2α and PGE2 are implicated in metastasis and migration [13,23,24], we next evaluated the effect of CIX on migration at dose levels that did not affect proliferation. The migratory capacity of the two cell lines were different, with 4T1 showing a five-fold higher basal migration as compared to 67NR after 24 h in normal controls (Figure 2a,c). The increased motility of the more aggressive 4T1 cells compared to 67NR cells is in agreement with previous reports [37].

Significant induction of migration in the positive controls (with increased levels of chemoattractant FBS) was demonstrated in both cell lines (Figure 2b,d). After 24 h, 7.5 or 15 µM CIX did not impair migration in 67NR; rather, 7.5 µM significantly increased migration of 67NR compared to the vehicle control. In contrast, CIX significantly inhibited migration in 4T1 in a dose dependent manner in both 24 h and 48 h experiments. For the 4T1 cells, the reduction of PGE2 and migration at the same CIX dose and time point may indicate that cPLA2α and PGE2 play a role in the increased migratory capacity of 4T1 relative to 67NR. However, the involvement of other eicosanoids and bioactive lipids such as lysophospholipids or platelet-activating factor cannot be ruled out. Other possible mechanisms where activation of cPLA2α facilitates migration include the generation of glycerophosphoinositol 4-phosphate, which activates Shp1 and Src and thus impacts actin remodeling in fibroblasts [38] and lysophosphatidic acid, as seen in ovarian cancer cells [39].

Pharmacological inhibition and gene silencing of cPLA2α has previously been shown to reduce migratory capacity in human breast cancer cell lines through the activation of epithelial-to-mesenchymal transition (EMT) [22]. The 4T1 model does not display a correlation between EMT phenotypic traits and aggressiveness [40], and therefore our findings may point to an EMT independent mechanism for the involvement of cPLA2α in migration. Previous reports have described signaling through PI3K/Akt and ERK in response to AA and AA metabolites as important for increased migration in breast cancer cells [21,41].

### 2.5. Transcriptomal Effects of cPLA2α Inhibition Include Toll-like Receptors and Type I Interferon Pathways

**Figure 3:**
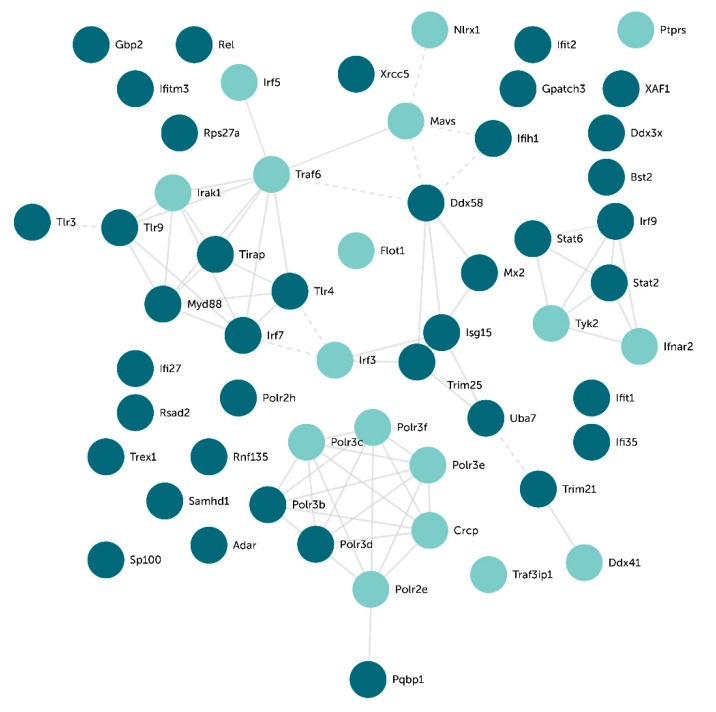
Gene clusters of top-ranked GO terms. Network of associated CIX-affected gene products were generated using STRING using only curated databases as active interaction sources. Light green nodes are upregulated, whereas dark green nodes are downregulated in 4T1 cells in response to CIX (15 µM, 24 h). TLR signaling stands out as a key cluster in this network. Solid lines represent intra-cluster edges, dotted lines represent inter-cluster edges. Solid lines represent intra-cluster edges, dotted lines represent inter-cluster edges.

**Figure A1:**
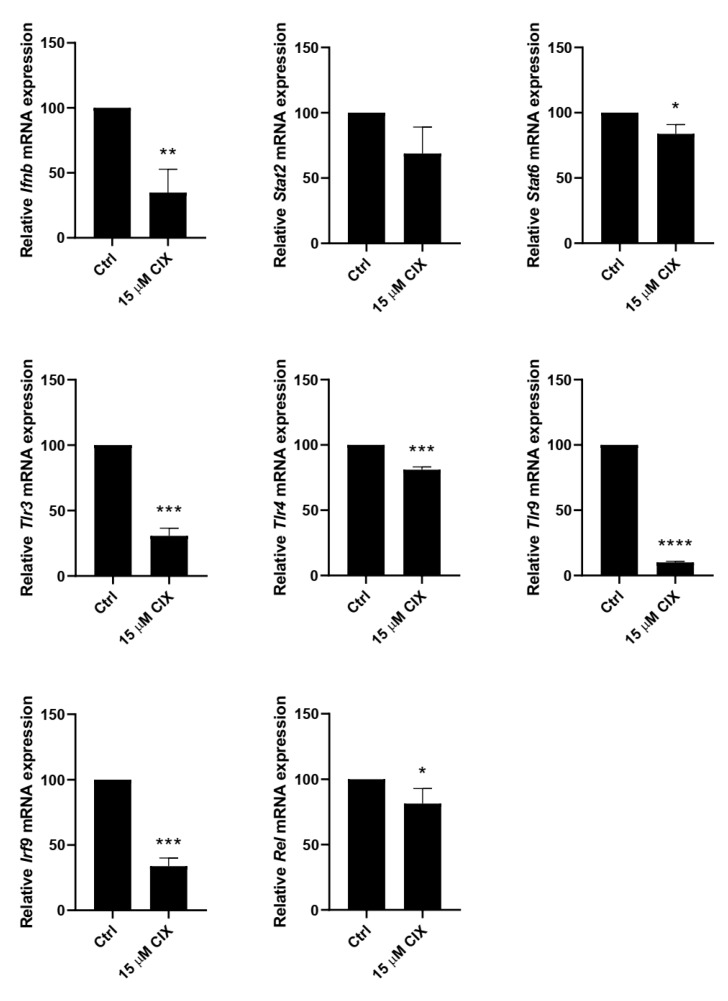
mRNA expression of *Ifnb, Stat2, Stat6, Tlr3, Tlr4, Tlr9, Irf9* and *Rel* in 4T1 cells treated with vehicle or 15 μM CIX for 24 h. Results shown are mean ± SD of four biologically independent experiments. * *p* < 0.05, ** *p* < 0.01, *** *p* < 0.001, **** *p* < 0.0001.

In order to investigate if the anti-migratory effect of CIX on 4T1 cells could be reflected in the transcriptome, we next performed a global gene expression analysis using RNA sequencing. In response to 15 µM CIX, 2887 genes were differentially expressed compared to untreated controls (see Appendix B for complete gene lists). To characterize the molecular responses affected by CIX treatment, the data set with differentially expressed genes were analyzed in Enrichr. The key Gene Ontology (GO) biological processes identified were related to type I interferon (IFN-I) and TLR signaling, RNA splicing, and cell cycle regulation (Table 1). Many of the genes associated with these processes were downregulated in CIX treated cells as compared to control cells (confirmed by RT-qPCR; Figure A1 in Appendix B). Next, using STRING, we made an interaction network with the genes in the top-ranked GO terms that were regulated by CIX treatment and linked to TLR or IFN-I signaling. This gave a network with several clusters, based on evidence of interaction between the proteins that are products of these genes (Figure 3).

One cluster appearing in our interaction network contained the TLRs, Tlr3, Tlr4, and Tlr9 and the adaptor proteins Tirap and Myd88, all significantly less expressed at the gene level in CIX treated 4T1 cells as compared to control cells. Furthermore, the TLR9-regulated transcription factor *IRF7*, inducing several IFN-α genes and cytokines, appeared in this cluster, and was downregulated in response to CIX. In contrast, the TLR3-regulated transcription factor *IRF3* was upregulated. TLR stimulation activates NF-κB, mitogen activated protein kinases (MAPKs), Jun N-terminal kinases (JNKs), p38, and ERKs, as well as interferon regulatory factors such as IRF3/7, in turn regulating the production of inflammatory cytokines [42]. Another prominent cluster contained the INF-I signaling pathway components STAT2, STAT6, IRF9, TYK2, and IFNAR2. Overall, it is difficult to interpret these findings, but our data suggests that Myd88-dependent TLR signaling is reduced in response to CIX, whereas the effect on MyD88-independent signaling is not known.

TLRs are central in recognition of invading microorganisms or internal damaged tissues, leading to an inflammatory response via the formation of inflammasome [3]. TLRs are also central in cancer, including breast cancer, and exert contrasting effects on immune cells versus cancer cells [43]. Using cPLA2α inhibitors, we have previously shown that cPLA2α regulates TLR2-induced PGE2 and proinflammatory gene expression in synoviocytes [29]. Both TLR4 and TLR9 signaling pathways are associated with increased migration in breast cancer [44,45,46]. A study by Wu et al. showed that the expression levels of TLR4 and MyD88 were significantly increased in breast tumors as compared with normal breast tissue. The expression levels of TLR4 and MyD88 were also positively correlated with the metastatic potential of breast cancer cells and tumors [47].

IFN-Is are induced by TLRs, which are pattern recognition receptors playing a pivotal role in innate immunity and cancer [48,49]. IFN-Is, like TLRs, are emerging as double-edged swords in cancer [50]. The role of IFN-Is in cancer has generally been considered beneficial by promoting T cell responses and preventing metastasis. On the other hand, IFN-Is may also have a negative role by promoting negative feedback and immunosuppression. IFN-I signaling may be a key driver of immune dysfunction in some cancers. Studies have shown an upregulation of the IFNα pathway in inflammatory breast cancer [51], and breast cancer tumors with high expression of interferon-response genes are shown to be associated with a significantly shorter overall survival and metastasis-free survival [52]. In our study, *STAT2*, *IRF9*, and *STAT6* are significantly less expressed in CIX treated cells as compared to control cells (confirmed by real-time quantitative PCR [RT-qPCR], Figure A1 in Appendix B), suggesting that cPLA2α inhibition interferes with IFN-I signaling in 4T1 cells. However, even though no IFN’s were themselves found to be regulated by CIX by RNA sequencing, RT-qPCR showed significantly less *INFBb* expression in CIX-treated cells (Figure A1, Appendix B), hence IFN levels should be investigated more closely. Loss of STAT2 has previously been associated with decreased proliferation and migration in triple-negative breast cancer [53].

**Figure 4:**
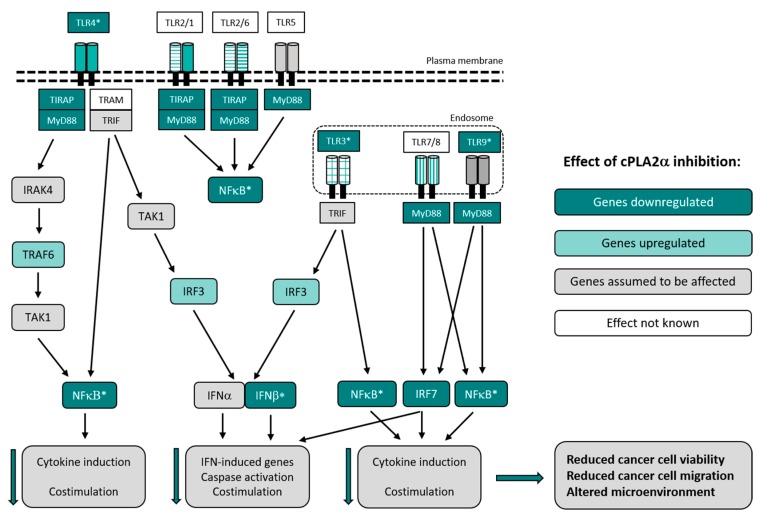
Hypothesized effects of CIX in TLR signaling in 4T1 cells. By reduced signaling through TLR3, TLR4, TLR9, and NF-κB (rel), it is likely that cPLA2α inhibition affects the cancer microenvironment and reduces both cancer cell viability and migration, and the level of inflammation. Such an altered cancer cell microenvironment may suggest that cPLA2α regulates many aspects of cancer cell biology and thus serves as an attractive target for metastatic cancer. The figure is modified after [47,54]. Dark green nodes represent genes found to be downregulated, light green nodes represent upregulated genes. Grey and white nodes represent genes assumed to be affected or without known effects, respectively. Downward pointing green arrows indicate that these processes are down-regulated by CIX. *Regulation of gene expression confirmed by RT-qPCR (Figure A1, Appendix B). Downward pointing green arrows indicate that these processes are down-regulated by CIX.

Together, this implies that cPLA2α inhibition, via reduction of PGE2 and TLRs and by interfering with IFN-I signaling, reduces migration in highly metastatic 4T1 cells (Figure 4).

Overall, our findings confirm that treatment with a low-molecular cPLA2α inhibitor reduces the viability of murine breast cancer cell lines. The metastatic 4T1 cell line had higher baseline cPLA2α activity and was less sensitive to CIX than the non-metastatic 67NR cell line metabolically, but more sensitive for the anti-migratory effect. cPLA2α inhibition reduced PGE2 production and blocked migration in the 4T1 cells. Gene expression analysis suggests the involvement of TLR and IFN-I signaling. Further research is needed to understand the mechanism of these observations and to evaluate the anti-metastatic effect of cPLA2α inhibition in vivo.

## 3. Materials and Methods

### 3.1. Cell Culture

Two isogenic cell lines, stemming from a single spontaneous mammary triple-negative tumor, were used for all experiments. While both cell lines effectively establish primary tumors, 67NR cells do not metastasize, and 4T1 cells can form metastatic lesions in lung, brain, lymph nodes, bone, and liver [32,37]. Cells were kept in vented flasks in a humidified atmosphere at 5% CO2, 37 °C, and stock flasks routinely split 1:8–1:10 (67NR) and 1:15–1:20 (4T1) twice a week using 0.25% trypsin/EDTA. Passage numbers were between 15 and 55. Culture medium was Dulbecco’s modified Eagle medium (DMEM)/4.5 mg/mL glucose (Gibco, Thermo Fisher Scientific, Waltham, MA, USA) with 10% fetal bovine serum (FBS; Gibco), 0.1 mg/mL penicillin-streptomycin, and 1 μg/mL amphotericin. Prior to experiments, stock cells were synchronized once or twice by seeding into a new flask with a split ratio of 1:2–1:5, incubated overnight, trypsinized and seeded into wells in growth culture medium. Seeded cells were allowed to attach and grow overnight before treatment with CIX [31]. Dimethyl sulfoxide (DMSO) was used as vehicle for the inhibitor in all experiments.

### 3.2. XTT Assay

The XTT (2,3-bis-(2-methoxy-4-nitro-5-sulfophenyl)-2H-tetrazolium-5-carboxanilide) assay is a colorimetric assay in which the tetrazolium salt is converted in mitochondria of metabolically active cells, hence the signal is proportional to viable cells [55]. Cells were seeded in 96-well flat-bottom plates, with cell numbers optimized to get 60% to 80% confluency at the start of the experiment. After overnight incubation, the growth medium was removed, and the experiment was performed in a serum-free medium. An incubation time of 48 h following addition of inhibitor was chosen for the readout in order to detect differential effects in subsequent assays. The TACS XTT Cell Proliferation/Viability Assay (Trevigen, Gaithersburg, MD, USA) was performed according to the manufacturer’s manual, with 1.5 to 2 h incubation. The readout at 490 nm with subtraction of background reading at 655 nm was carried out on an iMark Microplate Reader (BIO RAD, Hercules, CA, USA). Results were given as % viability based on the average of sextuplets as compared to the intra-assay vehicle controls. A two-tailed Student’s t-test was used to evaluate the statistical significance. IC50, the concentration which reduced signal to 50 % of controls, was calculated using non-linear fit of [Inhibitor] vs. response -- Variable slope (four parameters), in GraphPad 7 for Windows [56].

### 3.3. Proliferation Assay

A Click-IT EdU microplate assay (Invitrogen, Thermo Fisher Scientific) was used to determine the effect of CIX concentration on proliferation. Cells were seeded as described for the XTT assay. EdU (final concentration 3-5 μM) was added to wells directly after applying treatments, and cells were incubated for 24 h before performing the assay according to the manufacturer’s manual. Readout was carried out after 24 h EdU exposure on a POLARstar Omega plate reader (BMG LABTECH, Ortenberg, Germany) with excitation-emission at 540/580 with orbital averaging. The IC50 was calculated in the same way as for XTT data.

### 3.4. Protein Extraction

To generate protein lysate for subsequent assays, cells were seeded in 6-well plates to obtain 60% to 80% confluency 24 h post seeding. After treatment, supernatant was removed for subsequent ELISA, and proteins were extracted in ice-cold RIPA buffer containing 2% cOmplete™ protease inhibitor cocktail, 1% phosphatase inhibitor cocktail 2, and 1% phosphatase inhibitor cocktail 3 (all Merck KGaA, Darmstadt, Germany). The lysate was agitated for 30 min at 4 °C, sonicated for 1.5 min, and centrifuged for 20 min at 4 °C, 12,000 rpm. The protein supernatant was stored at −80 °C.

### 3.5. Western Blotting

Protein concentration was quantified using the Pierce BCA assay (Thermo Fisher Scientific) according to the manufacturer’s manual. Protein lysates were separated by sodium dodecyl sulfate polyacrylamide gel electrophoresis. IR-labeled secondary antibodies were used for Western blotting. All Bolt reagents were from Invitrogen, Thermo Fisher Scientific. Details on antibodies and blotting are provided in Appendix A1. Imaging was performed on an Odyssey Clx (LI-COR Biosciences, Lincoln, Nebraska, USA) in the 700 and 800 channels at the auto setting. The quantification of bands were performed by subtracting local background and the signal was normalized to beta actin in Image Studio Lite (LI-COR Biosciences). A two-tailed Student’s t-test was used to evaluate the statistical significance between groups.

### 3.6. Enzyme Immunoassay

A sensitive, quantitative enzyme immunoassay specific for the metabolite PGE2 was used as a proxy to assess cPLA2α activity. Supernatant from cells were spun at 1500 rpm for 10 min at 4 °C and stored at −80 °C. After thawing, supernatants were spun at 2000 rpm for 5 min at 4 °C, diluted 1:50 in DMEM, and analyzed in duplicate using a 96-well PGE2 EIA Kit Monoclonal (Cayman Chemical Company, Ann Arbor, MI, USA). The analysis was performed using the four parameter logistic curve function in the MyAssays Prostaglandin E2 Monoclonal online analysis tool [57]. A two-tailed Student’s t-test was used to evaluate the statistical significance (*p* < 0.05).

### 3.7. Migration Assay

Cell migration was assessed using the xCELLigence^®^ DP system (Roche Diagnostics GmbH, Mannheim, Germany). In this assay, cells are added to a membrane surface in the upper part of a CIM 16 plate, and cells can migrate through the membrane to the lower wells as a response to a chemoattractant signal. Gold electrodes are attached underneath the membrane, and cells in contact with electrodes reduce the conductivity. The change is interpreted inversely as the Cell Index (CI). Here, 5.0 × 10^4^ cells/well were seeded in 0.5% FBS/DMEM in upper wells containing inhibitor or vehicle (DMSO). As a chemoattractant, 5% or 10% FBS/DMEM was added to lower wells for normal or positive controls, respectively, with 0.5% FBS used to control for increased background migration (negative controls) in 48 h experiments. The plate was scanned every 15 min for 24 h or 48 h. The plotting of the CI curves was carried out using RTCA Software 1.2 (https://www.aceabio.com/products/rtca-dp/#tab-software) supplied with the instrument. For relative quantification, all technical replicates of a treatment were normalized to the intra-experiment normal control. A two-tailed Student’s t-tests were performed to find significant differences between groups (*p* < 0.05).

### 3.8. RNA Sequencing

RNA for sequencing of the global mRNA transcriptome was isolated from 4T1 or 67NR cells treated with 15 μM CIX or vehicle (*n* = 4 biological replicates) for 24 h, using the RNeasy Mini kit (Qiagen, Limburg, The Netherlands) according to the manufacturer’s manual. The RNA concentration and purity were quantified using a NanoDrop 1000 (Thermo Scientific, Waltham, USA). An Agilent Bioanalyzer (Agilent, Santa Clara, CA, USA) was used to measure RNA Integrity Numbers (RIN), which were between 9.7 and 10. A library was prepared using an Illumina TruSeq Stranded mRNA kit (Illumina, San Diego, CA, USA), sequencing was done on an Illumina NextSeq using a NS500HO flow cell with 75 bp reads, and quality control of raw sequences was performed with the FastQC application [58]. The data discussed in this publication have been deposited in NCBI’s Gene Expression Omnibus [59] and are accessible through GEO Series accession number GSE138026 (https://www.ncbi.nlm.nih.gov/geo/query/acc.cgi?acc=GSE138026)

### 3.9. Gene Expression Analysis

Transcript expression values were generated by quasi-alignment using salmon [60,61] and the Ensembl (GRCm38) mouse genome. Aggregation of transcript to gene expression was performed using tximport [62,63]. Gene expression values with TPM (transcript per million) below one in more than three samples were filtered out before differential expression was assessed by limma-voom [64,65] linear model. Significance was defined by a Benjamini–Hochberg multiple comparison-adjusted *p*-value < 0.001. Enrichr, a tool for gene enrichment analysis [66,67], was employed to find gene enrichment signatures for these genes within the Gene Ontology Biological Process 2018 database (geneontology.org). Significance was denoted as *p* < 0.05, corrected for multiple testing by the Benjamini–Hochberg procedure. Clustering of the proteins encoded by the genes included in the top-ranked GO terms, were performed using STRING version 11.0 [68] and modified using Adobe Illustrator (Adobe, San Jose, CA, USA).

### 3.10. RT-qPCR

The cDNA synthesis was performed with 1 µg total RNA in 20 µL reaction of QuantiTect Reverse Transcription Kit (Qiagen, Hilden, Germany). After synthesis, cDNA was diluted 1:6 with RNase-free water. The qPCR was performed with the LightCycler 480 SYBR Green I Master (Roche, Basel, Switzerland), and the qPCR analyses were carried out using the Lightcycler 96 system (Roche). *ACTB* was used as a reference gene. Primer sequences: *IFNB* forward; 5′-AACTTCCAAAACTGAAGACC-3′, reverse; 5′-AACTCTGTTTTCCTTTGACC-3′; *STAT2* forward; 5′-GACATTGAGTTCTTGGTGAG-3′, reverse; 5′-CAGACTGAAAACTGTGTATCTG-3′; *STAT*6 forward; 5′-CTGTTATAGAAGAGTTCCGC-3′, reverse; 5′-CATTGACAGGAGGGTCTATC-3′; *TLR3* forward; 5′-AATAGCATCAAAAGAAGCCG-3′, reverse; GATGTACCTTGAATCTTCTGC-3′; *TLR*4 forward; GATCAGAAACTCAGCAAAGTC-3′, reverse; 5′-TGTTTCAATTTCACACCTGG-3′; *TLR*9 forward; 5′-TCTCCCAACATGGTTCTC-3′, reverse; 5′-CTTCAGCTCACAGGGTAG-3′; *IRF*9 forward; 5′-CTACTTCTGTAGAGATTTGGC-3′, reverse; 5′-GATGAGATTCTCTTGGCTATG-3′; *REL* forward; 5′-ATACCTGCCAGATGAAAAAG-3′, reverse; 5′-TCAGTAAAGTGACCACAATC-3′; *ACTB* forward; 5′-GATGTATGAAGGCTTTGGTC-3′, reverse; 5′-TGTGCACTTTTATTGGTCTC-3′. Unpaired t-test with Welch’s correction was used to find significant differences between groups.

## 4. Conclusions

In this study, we provide evidence that the selective cPLA2α inhibitor, CIX, specifically impedes migration of metastatic 4T1 cells, but not of isogenic, non-metastatic 67NR cells. A comprehensive high throughput analysis at the transcriptome level in 4T1 cells indicates that cPLA2α inhibition affects TLR signaling and type I interferons.

## Figures and Tables

**Table 1:** GO biological processes terms. Enrichment analysis was performed using differentially expressed genes in treated vs. untreated 4T1 cells. Overlap signifies input genes that matched genes in GO term vs. total genes listed in GO term.

Term ID	Description	Overlap	False Rate Discovery
GO:0032648	regulation of interferon-beta production	18/35	0.000859
GO:0032481	positive regulation of type I interferon production	25/63	0.000859
GO:0032728	positive regulation of interferon-beta production	15/27	0.000859
GO:0032479	regulation of type I interferon production	31/86	0.000859
GO:0000398	mRNA splicing, via spliceosome	67/262	0.000993
GO:0006397	mRNA processing	71/284	0.000993
GO:0000377	RNA splicing, via transesterification reactions with bulged adenosine as nucleophile	62/237	0.000993
GO:0000086	G2/M transition of mitotic cell cycle	37/127	0.007793
GO:0044839	cell cycle G2/M phase transition	37/128	0.008405
GO:0060337	type I interferon signaling pathway	23/66	0.009802
GO:0071357	cellular response to type I interferon	23/66	0.009802
GO:0000070	mitotic sister chromatid segregation	27/83	0.009802
GO:0018205	peptidyl-lysine modification	33/116	0.022896
GO:0051310	metaphase plate congression	17/45	0.027307
GO:0010506	regulation of autophagy	50/204	0.027307
GO:0044772	mitotic cell cycle phase transition	53/222	0.03186
GO:0002181	cytoplasmic translation	19/55	0.038663

## Abbreviations

AA	Arachidonic acid
CIX	cPLA2α inhibitor X
COX2	Cyclooxygenase-2
DMEM	Dulbecco’s modified eagle medium
DMSO	Dimethyl sulfoxide
EMT	Epithelial-to-mesenchymal transition
FBS	Fetal bovine serum
GO	Gene ontology
IFN	Interferon
NF-κB	Nuclear factor kappa B
PGE2	Prostaglandin E2
TLR	Toll-like receptor

## Appendix A

### Experimental Details for Western Blotting

The protein lysate was heated at 70 °C for 10 min in 4X Protein Loading Buffer (LI-COR Biosciences, Lincoln, NE, USA) and Bolt Sample Reducing Agent, before 5 μg protein was loaded onto a Bolt Bis-Tris 4% to 12% gel and separated at 200 V for 70 min, using Bolt MOPS SDS running buffer and Bolt Antioxidant in front chamber and Chameleon Duo Pre-stained Protein Ladder (LI-COR Biosciences) for size marking. Wet transfer to PVDF membrane was done at 20 V for 2 h, using Bolt Transfer buffer. Following blocking with Odyssey Blocking buffer TBS (LI-COR Biosciences) overnight at 4 °C and cutting at 50 kDa mark for separate incubation with anti-beta actin antibodies, the membranes were incubated with goat anti-phospholipase A2 antibody (ab104252, Abcam; 1: 3000) and rabbit anti-phospholipase A2 (phospho S505) antibody (ab53105 from Abcam, Cambridge, UK; 1:3000), or mouse anti-beta actin (ab8226 from Abcam; 1:25,000), overnight at 4 °C. Secondary fluorescent antibody conjugates (IRDye^®^ 800CW donkey anti-Goat IgG and 680CW Goat anti-rabbit IgG, or IRDye^®^ 800CW Goat Anti-mouse IgG; LI-COR Biosciences) were applied at 1:30,000 dilution in blocking buffer/0.2% Tween-20/0.01% SDS.

## Appendix B

**Table A1:** Gene lists for GO Biological Processes terms. The listed genes are differentially expressed genes in treated vs. untreated 4T1 cells overlapping with genes in the GO term lists as analyzed with Enrichr [66,67].

Term ID	Genes
GO:0032648	*PTPRS;DDX3X;DDX58;TIRAP;IFIH1;RNF135;MAVS;IRF3;POLR3B;POLR3C;POLR3D;FLOT1;TLR9;POLR3F;IRF7;REL;TLR4;TLR3*
GO:0032481	*CRCP;DDX3X;DDX41;PQBP1;IFIH1;RNF135;IRAK1;FLOT1;POLR2E;STAT6;POLR2H;XRCC5;DDX58;MAVS;IRF3;POLR3B;POLR3C;POLR3D;POLR3E;TLR9;POLR3F;IRF7;TLR4;TLR3;MYD88*
GO:0032728	*DDX3X;DDX58;IFIH1;RNF135;MAVS;IRF3;POLR3B;POLR3C;POLR3D;FLOT1;TLR9;POLR3F;IRF7;TLR4;TLR3*
GO:0032479	*NLRX1;CRCP;UBA7;TRAF3IP1;GPATCH3;DDX41;PQBP1;IFIH1;RNF135;IRAK1;POLR2E;TRIM25;STAT6;POLR2H;RPS27A;TRIM21;XRCC5;DDX58;TREX1;ISG15;TIRAP;MAVS;IRF3;POLR3B;POLR3C;POLR3D;POLR3E;POLR3F;IRF7;MYD88;TRIM56*
GO:0000398	*DBR1;TXNL4B;DDX46;HNRNPU;WDR83;CWC25;YBX1;DDX41;PQBP1;CCAR1;CLP1;RSRC1;MAGOH;DHX16;SNRNP35;SRRM2;RBM17;PRMT7;BUD31;METTL3;PRPF40B;PLRG1;CDC40;WDR77;SRRM1;SFPQ;HNRNPUL1;FRG1;GEMIN5;NAA38;SNRPA1;HNRNPH2;PABPC1;SRSF6;SNRPB;RBM22;SRSF9;ZCCHC8;DDX1;PRCC;SRRT;DDX23;SRSF1;CSTF2T;RNPC3;HTATSF1;PRPF8;RBM15B;PUF60;PCBP1;TRA2A;ZMAT5;POLR2E;POLR2G;POLR2H;HNRNPA1;DCPS;SRSF10;HNRNPA3;SF3A1;CPSF7;PRPF38A;CDC5L;LSM2;HNRNPM;LSM6;RBM41*
GO:0006397	*DBR1;DDX46;HNRNPU;WDR83;CWC25;YBX1;DDX41;PQBP1;CCAR1;CLP1;RSRC1;MAGOH;DHX16;SNRNP35;CMTR2;SRRM2;RBM17;BUD31;METTL3;THOC1;PRPF40B;PLRG1;CDC40;SRRM1;SFPQ;PRPF18;HNRNPUL1;FRG1;GEMIN5;NAA38;SNRPA1;HNRNPH2;PABPC1;SRSF6;SNRPB;RBM22;SRSF9;ZCCHC8;PRCC;SRRT;DDX23;SRSF1;CSTF2T;RNPC3;HTATSF1;PRPF8;RBM15B;PUF60;PCBP1;TRA2A;ZMAT5;POLR2E;POLR2G;POLR2H;HNRNPA1;SRSF10;HNRNPA3;SF3A1;CPSF7;CHTOP;PRPF38A;CPSF6;CDC5L;GTF2H1;LSM2;HNRNPM;CDK7;LSM6;RBM41;AGGF1;MNAT1*
GO:0000377	*DBR1;DDX46;HNRNPU;WDR83;CWC25;YBX1;DDX41;PQBP1;CCAR1;CLP1;RSRC1;MAGOH;DHX16;SNRNP35;SRRM2;RBM17;BUD31;METTL3;PRPF40B;PLRG1;CDC40;SRRM1;SFPQ;HNRNPUL1;FRG1;GEMIN5;NAA38;SNRPA1;HNRNPH2;PABPC1;SRSF6;SNRPB;RBM22;SRSF9;ZCCHC8;PRCC;SRRT;DDX23;SRSF1;CSTF2T;RNPC3;HTATSF1;PRPF8;RBM15B;PUF60;PCBP1;TRA2A;ZMAT5;POLR2E;POLR2G;POLR2H;HNRNPA1;SRSF10;HNRNPA3;SF3A1;CPSF7;PRPF38A;CDC5L;LSM2;HNRNPM;LSM6;RBM41*
GO:0000086	*DYNC1I2;CDKN1A;DCTN2;DCTN1;SSNA1;CEP164;FOXM1;CCNB2;PPME1;CNTRL;RPS27A;LCMT1;CEP290;CEP76;PLK3;BORA;ODF2;CEP152;MBD1;PLK1;FBXL15;HAUS5;MASTL;DYNLL1;CDC25C;CDC25B;DNM2;HAUS1;CCNA2;CDK7;CENPJ;CDK2;MAPRE1;MNAT1;SFI1;TAF2;SDCCAG8*
GO:0044839	*DYNC1I2;CDKN1A;DCTN2;DCTN1;SSNA1;CEP164;FOXM1;CCNB2;PPME1;CNTRL;RPS27A;LCMT1;CEP290;CEP76;PLK3;BORA;ODF2;CEP152;MBD1;PLK1;FBXL15;HAUS5;MASTL;DYNLL1;CDC25C;CDC25B;DNM2;HAUS1;CCNA2;CDK7;CENPJ;CDK2;MAPRE1;MNAT1;SFI1;TAF2;SDCCAG8*
GO:0060337	*IFNAR2;IFITM3;SP100;RSAD2;STAT2;MX2;ADAR;ISG15;TYK2;IFI35;SAMHD1;IFIT1;IFIT2;ISG20;BST2;IRF3;IRAK1;IFI27;IRF7;GBP2;XAF1;MYD88;IRF9*
GO:0071357	*IFNAR2;IFITM3;SP100;RSAD2;STAT2;MX2;ADAR;ISG15;TYK2;IFI35;SAMHD1;IFIT1;IFIT2;ISG20;BST2;IRF3;IRAK1;IFI27;IRF7;GBP2;XAF1;MYD88;IRF9*
GO:0000070	*SEH1L;PHF23;VPS4A;PINX1;CDCA8;SMC4;NSL1;NCAPH;SMC2;CHMP1B;RRS1;DLGAP5;DIS3L2;NAA50;SPAG5;BOD1;PLK1;TUBG2;KNSTRN;PSRC1;KIF18B;KIFC1;CHMP2A;KIF2C;NCAPD2;CHMP7;CHMP5*
GO:0018205	*HDAC4;TOP2B;POM121;NUP205;PCNA;SEH1L;CDCA8;LOXL4;PLOD2;PLOD1;AAAS;AURKB;IFIH1;SENP6;MTA1;SUMO1;SENP8;NFATC2IP;NUP43;NDC1;PIAS3;ESCO1;SIRT4;MITF;RANGAP1;TOPORS;SIRT2;INCENP;CTH;NDUFAB1;NUP35;BIRC5;VIPAS39*
GO:0051310	*SEH1L;BOD1;VPS4A;PINX1;CDCA8;GEM;CENPF;PSRC1;KIFC1;CHMP1B;RRS1;CHMP2A;KIF2C;FAM83D;CHMP7;CENPQ;CHMP5*
GO:0010506	*GSK3A;CISD2;ZDHHC8;CAPNS1;MLST8;WDR6;PIP4K2C;DNM1L;ATP6V1E1;TRIM21;TRIM65;SREBF1;RUBCN;EXOC8;CSNK2A2;SIRT2;MID2;RRAGA;KAT2A;CPTP;ATP6V1B2;ULK3;ULK2;SMCR8;ATP6V1A;GBA;HTRA2;VPS26A;RASIP1;MAPK8;DHRSX;LARS;ATG101;ATP6V1H;SH3BP4;BMF;SLC38A9;ATP6V0E2;ATP6V1D;SNX5;ATP6V1C1;PLK2;TPCN1;CDK5;BCL2;LAMTOR2;LAMTOR4;LAMTOR3;CDK5R1;LAMTOR5*
GO:0044772	*CDKN1A;CEP164;FOXM1;PPME1;MYC;CNTRL;CEP290;BORA;HAUS5;MASTL;DYNLL1;CDC25C;CDC25B;DNM2;HAUS1;CCNA2;CCNE2;MAPRE1;SFI1;CHMP7;CACUL1;ANAPC1;SDCCAG8;DYNC1I2;DCTN2;DCTN1;SSNA1;PRIM1;CUL2;ORC5;CCNB2;RPS27A;LCMT1;CEP76;CDT1;PLK3;ODF2;UBE2C;CEP152;PLK2;MBD1;PLK1;FBXL15;CDK7;POLA2;CDK6;CENPJ;POLE3;CDK2;TACC3;CHMP2A;MNAT1;TAF2*
GO:0002181	*RPL4;RPL32;RPLP1;RPL22;RPL7;RPS26;RPL18A;RPS29;RPL27A;MRTO4;RPS3;RPL36;RPLP2;RPL24;RPL22L1;RPL15;RPL29;EIF4B;RPL19*
